# Transcriptome Changes in Relation to Manic Episode

**DOI:** 10.3389/fpsyt.2019.00280

**Published:** 2019-05-01

**Authors:** Ya-Chin Lee, Yu-Lin Chao, Chiao-Erh Chang, Ming-Hsien Hsieh, Kuan-Ting Liu, Hsi-Chung Chen, Mong-Liang Lu, Wen-Yin Chen, Chun-Hsin Chen, Mong-Hsun Tsai, Tzu-Pin Lu, Ming-Chyi Huang, Po-Hsiu Kuo

**Affiliations:** ^1^Institute of Epidemiology and Preventive Medicine, College of Public Health, National Taiwan University, Taipei, Taiwan; ^2^Department of Psychiatry, Buddhist Tzu Chi General Hospital and Tzu Chi University, Hualien, Taiwan; ^3^Department of Psychiatry, National Taiwan University Hospital, Taipei, Taiwan; ^4^Department of Psychiatry, Wang-Fang Hospital, Taipei Medical University, Taipei, Taiwan; ^5^Department of Psychiatry, School of Medicine, College of Medicine, Taipei Medical University, Taipei, Taiwan; ^6^Department of Psychiatry, Taipei City Psychiatric Center, Taipei City Hospital, Taipei, Taiwan; ^7^Institute of Biotechnology, National Taiwan University, Taipei, Taiwan; ^8^Department of Public Health, National Taiwan University, Taipei, Taiwan

**Keywords:** bipolar disorder, manic episode, microarray, RNA-sequencing, transcriptome, noncoding RNAs, state markers

## Abstract

Bipolar disorder (BD) is highly heritable and well known for its recurrent manic and depressive episodes. The present study focused on manic episode in BD patients and aimed to investigate state-specific transcriptome alterations between acute episode and remission, including messenger RNAs (mRNAs), long noncoding RNAs (lncRNAs), and micro-RNAs (miRNAs), using microarray and RNA sequencing (RNA-Seq) platforms. BD patients were enrolled with clinical information, and peripheral blood samples collected at both acute and remission status spanning for at least 2 months were confirmed by follow-ups. Symptom severity was assessed by Young Mania Rating Scale. We enrolled six BD patients as the discovery samples and used the Affymetrix Human Transcriptome Array 2.0 to capture transcriptome data at the two time points. For replication, expression data from Gene Expression Omnibus that consisted of 11 BD patients were downloaded, and we performed a mega-analysis for microarray data of 17 patients. Moreover, we conducted RNA sequencing (RNA-Seq) in additional samples of 7 BD patients. To identify intraindividual differentially expressed genes (DEGs), we analyzed data using a linear model controlling for symptom severity. We found that noncoding genes were of majority among the top DEGs in microarray data. The expression fold change of coding genes among DEGs showed moderate to high correlations (∼0.5) across platforms. A number of lncRNAs and two miRNAs (*MIR181B1* and *MIR103A1*) exhibited high levels of gene expression in the manic state. For coding genes, we reported that the taste function-related genes, including *TAS2R5* and *TAS2R3*, may be mania state-specific markers. Additionally, four genes showed a nominal *p*-value of less than 0.05 in all our microarray data, mega-analysis, and RNA-Seq analysis. They were upregulated in the manic state and consisted of *MS4A14*, *PYHIN1*, *UTRN*, and *DMXL2*, and their gene expression patterns were further validated by quantitative real-time polymerase chain reaction (PCR) (qRT-PCR). We also performed weight gene coexpression network analysis to identify gene modules for manic episode. Genes in the mania-related modules were different from the susceptible loci of BD obtained from genome-wide association studies, and biological pathways in relation to these modules were mainly related to immune function, especially cytokine–cytokine receptor interaction. Results of the present study elucidated potential molecular targets and genomic networks that are involved in manic episode. Future studies are needed to further validate these biomarkers for their roles in the etiology of bipolar illness.

## Introduction

Bipolar disorder (BD) is a severe and highly heritable psychiatric disorder, characterized by repeated manic episodes and depressive episodes (1). Having the mania episode is a unique feature for diagnosing BD. The symptoms of mania episode include elevated mood, irritability, racing thoughts and rapid speech, inflated self-esteem, increased activity, reduced need for sleep, and engaged in risky behaviors (2). BD is among the top list of disease burden worldwide and causes huge loss of disability-adjusted life year (3). However, the pathological mechanisms of BD are still unclear. It remains a great challenge to make accurate and early diagnosis, as well as efficient treatment for BD (4). In the past decade, genome-wide association studies (GWAS) had huge impact on studying complex traits (5) and facilitated the identification of hundreds of genetic loci (6) and follow-up functional studies (7). However, the progress made for BD is left behind (8). In particular, the mechanisms underlying episodic feature of BD are largely unknown. In this regard, the dynamic characteristics of transcriptome that are in response to physiological and environmental stimuli become a suitable genomic system to study the molecular alterations for manic episode.

Recently, with the advances in RNA-sequencing (RNA-Seq) technology (9) and breakthrough findings of noncoding RNAs (10–12) (ncRNAs), studies in transcriptomics have entered a new era. Among different categories of ncRNAs, short micro-RNAs (miRNAs) are probably the most studied, with many known molecular functions, including binding mechanism and target gene repression. Another novel group of large ncRNAs, named long noncoding RNAs (lncRNAs) that are defined as transcripts longer than 200 bps, have been identified and found to have substantial regulation functions during development in multiple genomic domains (13), including transcriptional and posttranscriptional regulations (10, 11). LncRNAs exert their complex functions by further interacting with miRNAs as competitors, primers, or cooperators to regulate miRNAs or other regulators (12). Moreover, lncRNAs are vital for brain development and neural function (14), and are recognized to play roles in psychiatric disorders (15). For example, the lncRNA myocardial infarction associated transcript (MIAT) (Ensembl gene ID: ENSG00000225783) has been found to regulate schizophrenia-associated alternative splicing and is regulated by neuronal activation (16). Akula et al. conducted the first RNA sequencing (RNA-Seq) analysis in postmortem brains of 11 BD and 8 healthy controls to explore between-group transcriptome differences (17). Together with results from another study in BD (18), it was reported that lncRNAs were significantly expressed with difference. Moreover, using human induced pluripotent stem cells (iPSCs), RNA-Seq result showed dysregulated lncRNAs in BD patients compared to healthy control iPSCs (19). These results highlight the importance to study both coding and noncoding transcriptomic targets for psychiatric disorders.

It is known that transcriptome profiles are different across individuals and are easily confounded by environmental and genetic factors, RNA extraction methods, and sample timing (20, 21). Therefore, it might be more appropriate to study within-individual transcriptomic differences such as disease state markers to reduce potential confounding effects by interindividual comparisons. The concept of studying state-specific markers for BD using gene expression data is not new (22). For example, brain-derived neurotrophic factor, the *BDNF* gene, is widely studied for its association with disease status of BD (23). Previous studies often adopted a candidate gene approach and used quantitative real-time PCR to investigate gene expressions in specific candidate genes (24) but not at the genome-wide scale to explore more comprehensive transcriptome alterations for mood episodes. In addition, despite a few studies exploring gene expression patterns for depressive states, very few studies were conducted for the manic state of BD (22), and none reported results of within-individual comparisons. As far as we are aware, there was only one study using prospective study design to follow 11 BD patients from manic episode to euthymia to explore blood gene expression alterations (25). However, this study reported results from inter-individual comparisons, including mania patients versus healthy controls, and euthymia patients versus healthy controls.

In the present study, we aimed to investigate transcriptome patterns for the unique manic feature of BD patients, including coding genes and ncRNAs. We followed patients from acute episode to remission and made intraindividual comparisons. The current study included discovery samples and replication samples using different high-throughput platforms of gene expression for comparisons. We also accessed public data from Witt et al. (25) to run a mega-analysis with their 11 pairs of BD samples in order to obtain more robust results combining different studies and ethnic groups. To identify differentially expressed genes (DEGs), we performed analyses using linear regression models adjusted for symptom severity. Using the latest gene annotation version, we were able to annotate all recorded ncRNAs in the database to obtain a more comprehensive transcriptome profile for the manic state. Moreover, the field is experiencing a paradigm shift for considering “omnigenic model” (26) and network analysis (27). We performed coexpression and network analyses to explore modules/pathways that are correlated with manic episode and symptom severity. These strategies could help us to better interpret the potential functions of identified coding genes and ncRNAs, and facilitate our understanding about the underlying mechanisms for the development of manic episode.

## Material and Methods

### Subjects

Inpatients aged 20–65 years old who were diagnosed with BD and had a current manic episode according to the criteria of *Diagnostic and Statistical Manual of Mental Disorders, Fourth Edition* were referred by psychiatrists in several central and regional hospitals in Taipei. We excluded patients with mental retardation, schizophrenia, schizoaffective disorder, or substance-induced secondary BD. Acute manic patients were followed for at least 2 months until they achieved full remission. Each patient had pair data with two time points, including clinical data and bio-sample collection. In total, we recruited 13 BD patients with manic episode, who had complete data in acute phase and remission status in the present study. Six BD patients were discovery samples using genome-wide microarrays, and seven BD patients undergone whole-genome RNA-Seq analysis. All participants signed informed consent forms after study procedures were fully explained. The sample recruitment and data collection were approved by the Institutional Review Board of all participating institutes and hospitals.

For replication, we further downloaded the microarray gene expression data of Witt et al. (25) with 11 German BD patients from Gene Expression Omnibus database (GEO, Series: GSE46416), which included data from both manic and remission status, and obtained patients’ clinical severity information from the corresponding author. The demographic data of all subjects were listed in **Table 1**. We conducted a mega-analysis to combine the two sets of microarray gene expression data as replication.

**Table 1:** Demographic data of all subjects in different platforms of the present study.

	Microarray	RNA-Seq	Witt’s study
**Samples**	6	7	11
**Male (%)**	3 (50)	4 (57)	11 (100)
**Age (mean ± SD)**	45.2 ± 9.9	42.6 ± 13.0	48.3 ± 12.0
**YMRS in acute state (mean ± SD)**	30.2 ± 5.8	24.3 ± 6.4	25.2 ± 10.2
**YMRS in remission (mean ± SD)**	4.7 ± 2.9	2.1 ± 2.5	4.3 ± 4.7
**HAMD in acute state (mean ± SD)**	7.7 ± 2.7	4.7 ± 3.0	N/A
**HAMD in remission (mean ± SD)**	4.3 ± 1.2	1.3 ± 1.3	N/A

The Microarray column was our primary analysis with Affymetrix Human Transcriptome Array (HTA) 2.0 and Witt’s study using Affymetrix Exon 1.0 ST array. For RNA sequencing (RNA-Seq), we used Illumina NextSeq 500 platforms. YMRS, Young Mania Rating Scale; HAMD, Hamilton Rating Scale, N/A, not applicable.

### Assessment

Our trained lay interviewers conducted a face-to-face interview with each participant. Subjects were interviewed with modified Chinese version of Schedule for Affective Disorders and Schizophrenia-Lifetime (SADS-L) to assess demographic characteristics and lifetime history of psychiatry disorders (28). For symptom severity, we used the Chinese version of Young Mania Rating Scale (YMRS) for mania (29) and its reliability, validity and sensitivity are examined. There was a high correlation between the scores of two independent clinicians on both the total score (0.93 and Hamilton Rating Scale (HAMD) for depression (30). A cutoff of 16 in YMRS score was set for defining individuals in manic episode, while a score of less than 7 was considered in remission. We defined a variable named stage to represent the contrast between acute and remission status. The HAMD score was less than 11 in all times, and this criterion was taken to ensure that the BD patients were in mania but not mixed state.

### RNA Isolation and Biosample Quality Control

The whole blood samples were drawn from patients at each time point and stored with TRizol^™^ reagent at −80°C freezers. We used chloroform for lysis, followed by isopropanol with incubation and centrifugation to retrieve total RNA precipitates from mononuclear cells. Then, we used 70% ethanol for washing. Finally, we solubilized the RNA pellet in 20–50 μL of RNase-free water and 0.1 mM EDTA for further application. RNA samples had undergone quality controls with the following criteria: OD260/280 was between 1.8 and 2.0, and OD260/230 was larger than 2.0. The quality control criteria of BioAnalyzer to obtain the RNA integrity number (RIN) and 28S/18S values were as follows: RIN ≥ 6 and 28S/18S ≥ 1.0. Most of our sequenced samples were of fine RNA quality. The mean of the RIN in all samples was 8.8, with only two samples with RIN values ranging from 6 to 7 (see **Supplementary Table S1**).

### Microarrays and RNA Sequencing

For transcriptome analysis, we conducted the genome-wide gene expression analysis using microarrays in the discovery samples. We used Affymetrix Human Transcriptome Array (HTA) 2.0, which contained more than 6.0 million probes and can be annotated to 44,699 coding transcripts and 22,829 noncoding transcripts, including miRNAs and lncRNAs. We used qualified 500 ng RNAs to be synthesized to cDNA and hybridized to the HTA microarrays. All procedures followed the Affymetrix protocols and performed in the Microarray Core Lab of Core Instrument Center in the National Health Research Center. The transcriptome microarrays used by Witt et al. (25) were Affymetrix Human Exon 1.0 ST Array, which contained 46,753 transcripts in total. For RNA-Seq, the qualified RNAs were used for library construction and then sequenced on Illumina NextSeq 500 platform with 75 paired-end sequencing. On average, each sample had more than 30 million reads.

### Transcriptome Analysis

All of the transcriptome analyses for microarrays were conducted with R software (31). First, the microarray data were imported and normalized using Robust Multichip Average (RMA) method (32), including background correction, log2 transformation, and quantile normalization, in *affy* package (33). To obtain the systematical nomenclature for cross-platform comparisons between different arrays, we used *biomaRt* package (34) to match probe ID in different microarray platforms with Ensembl gene ID using Human Genome Reference GRCh38 (35). Next, we used the collapseRows MaxMean function in *weighted gene co-expression network analysis (WGCNA)* package to select the represented probe expression value in gene-level comparison and increase between-study consistency. The MaxMean function was originally developed to perform cross-platform microarray mega-analysis and has been extensively validated (36). In total, the common genes between different platforms for the further analysis were 34,576 genes, including 18,049 coding genes and 16,527 noncoding genes. Finally, the possible batch effects were corrected with ComBat function of *sva* package (37). To capture the DEGs for between manic episode and remission, we constructed a linear model with the following covariates: age, sex, and YMRS score by *limma* package (38) to calculate expression values with empirical Bayes methods. For RNA-Seq, we performed analyses in linux system. We first used FastQC (https://www.bioinformatics.babraham.ac.uk/projects/fastqc/) for quality check for sequence files and used trimmomatic (39) for sequence trimming. We then used HISAT2 (40) for sequence alignment with GRCh38 index files and used featureCounts (41) to do gene annotations with Ensembl gene ID. Lastly, we used DESeq2 (42) to detect DEGs for intraindividual comparisons. We reported DEGs with a nominal *p*-value of <0.05 and an absolute value of fold change larger than 1.2.

### Quantitative Real-Time PCR for Potential Targets

For experimental validation, we used 10 pairs of RNA samples from manic episodes and remission. Among the 10 paired samples, 4 paired samples were technical replicates from previous samples and 6 paired samples were additional (see **Supplementary Table S2**).

For qRT-PCR, we performed standard RNA isolation and reverse transcription (RT) using PrimeScript RT reagent Kit (TaKaRa, Japan) following the manufacturer’s protocol. The analysis was conducted with SYBR^®^ Premix Ex Taq^™^ (TaKaRa, Japan), with sequence-specific primers of each targeting genes (see **Supplementary File** for all the target sequences). The qPCR assays were conducted in duplicate by MyiQ^™^ single-color real-time PCR detection system (Bio-Rad, CA), and glyceraldehyde 3-phosphate dehydrogenase (GAPDH) was used as internal control. Threshold was set above nontemplate control background and within linear phase of target gene amplification to calculate cycle number at which the transcript was detected [threshold cycle (CT)].

### Network and Enrichment Analyses

We ran weighted gene coexpression network analysis using the *WGCNA* package (43) for 17 BD samples altogether, and the two-time point data were normalized and batch effects were corrected before analysis. We used signed hybrid network type and biweight midcorrelation (bicor) for module detection and soft-threshold power calculation, which could provide robust results with better biological meanings (44). A soft-threshold power of 4 could achieve approximate scale-free topology with *R* ^2^ > 0.8. The network construction was created using blockwiseModules function for consideration of computer efficiency. The module detection criteria were as follows: minimum module size of 50, deepsplit of 4, and merge threshold of 0.25. The merged modules were then summarized with module eigengene (ME) correlations >0.75. MEs were defined by their first principal component and were labeled with different colors as module names in the Results section. After the modules were generated, we conducted different enrichment analysis to explore the functional interpretation of genes within the modules.

Moreover, we conducted enrichment analysis with genome-wide association (GWA) signals for BD. We downloaded the summary statistics results from one of the latest GWA study of BD from the Psychiatric Genomic Consortium from BioRxiv (8). We used loci with significant level less than a *p*-value of 5*10^−6^ and reannotated them with Ensembl database (35). In total, we obtained 131 genes, including coding and noncoding genes for the GWA enrichment analysis. There were, in total, 137 DEGs that showed signals in both our discovery samples and mega-analysis. We thus conducted DEG enrichment analysis to explore whether the mania-related modules are more enriched with these DEG signals. The *p*-value of each module for enrichment analyses was calculated using Fisher’s exact test. Lastly, for modules significantly associated with mania status or YMRS scores, we conducted gene-set analysis for genes in these identified modules to explore their functions using Multi-marker Analysis of GenoMic Annotation (MAGMA) (45). The background pool of gene-set analysis contained all the 34,576 genes as reference. To explore tissue specificity for these modules, we used data from GTEx project (46) and BrainSpan Atlas (47). The significance criterion was set with an adjusted *p*-value of <0.05.

## Results

**Figure 1:**
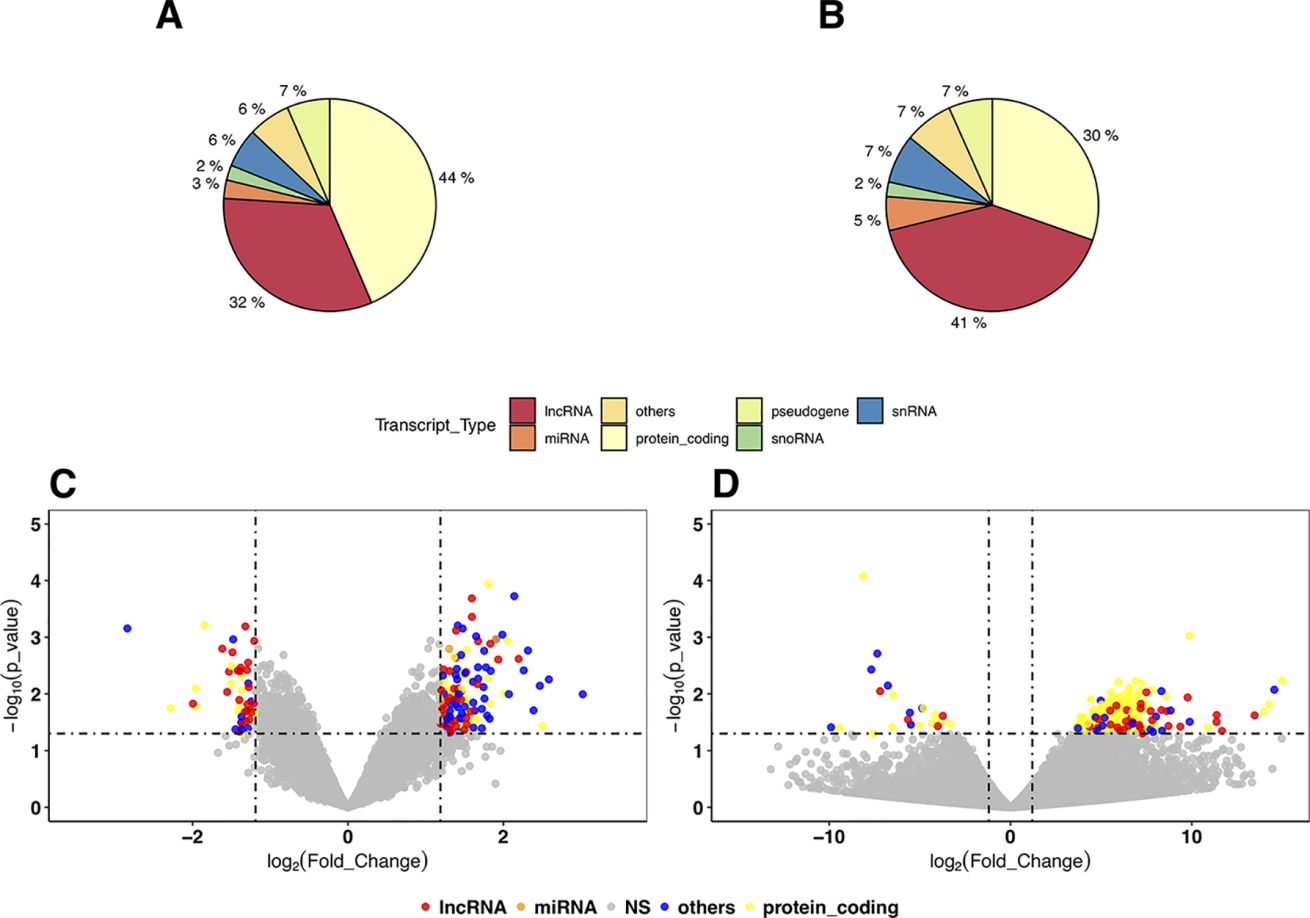
The distribution and volcano plot of differentially expressed genes (DEGs). The color in all cells: blue for protein coding genes, pink for long noncoding RNAs (lncRNAs), green for microRNAs, and purple for other RNA genes (small nuclear RNAs, ribosomal RNAs, small noncoding RNAs, etc). **(A)** The distribution of DEGs in the discovery samples with Human Transcriptome Array (HTA) 2.0. **(B)** The distribution of DEGs in mega-analysis of all microarray samples. **(C)** The volcano plot of mega-analysis of all microarray samples. **(D)** The volcano plot of RNA sequencing (RNA-Seq) results.

**Figure 2:**
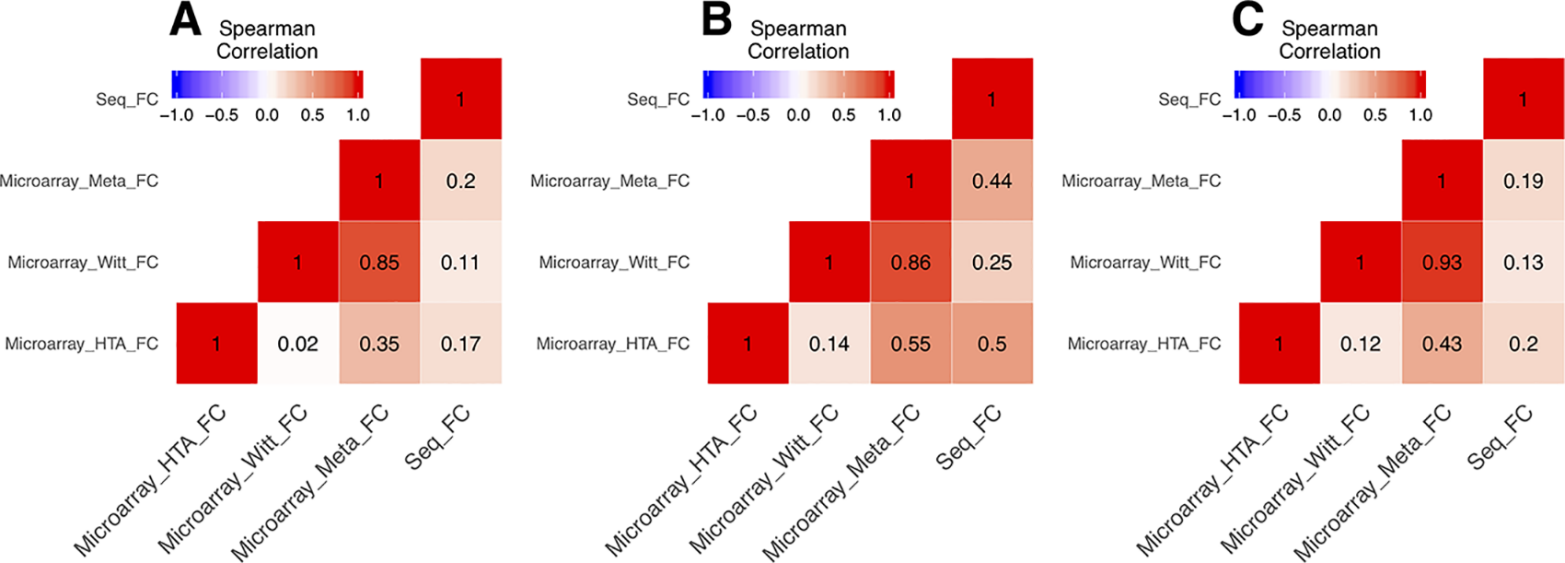
The correlations of fold change between microarrays and RNA-Seq. All the correlations were conducted with Spearman’s correlation. **(A)** The correlations of fold change of all genes between microarrays and RNA-Seq. **(B)** The correlations of fold change of the coding genes among union DEGs between microarrays and RNA-Seq. **(C)** The correlations of fold change of the noncoding genes among union DEGs between microarrays and RNA-Seq.

In the DEG analysis, we constructed a linear model with YMRS score in the model for intraindividual comparisons between episodic status. Results of the DEG analysis showed reasonable consistency between DEGs obtained from either stage or symptom severity. The correlations between them were high, whether in our discovery samples or in Witt et al. (25) samples, with a correlation coefficient of around 0.69–0.84 (see **Supplementary Figure 1**). We found that most of the DEGs were upregulated in the manic state (see **Figure 1C and D** and **Table 2**). There were 306 DEGs in our discovery microarray data, and 321 DEGs in the mega-analysis. Among the 306 DEGs in the discovery data, 60 DEGs remained significant in the mega-analysis, and the predominant gene category was ncRNAs (more than 40%, see **Figure 1A and B**). However, none of the DEGs meet statistical significance criterion after multiple testing correction, regardless of DEGs from discovery or mega-analysis. We further evaluated the gene expression correlations across experimental platforms. The correlations for the fold change between HTAs, RNA-Seq, and mega-analysis were shown in **Figure 2**, with a significant and modest correlation between microarray (mega-analysis) and RNA-Seq (0.20 in **Figure 2A**). The correlations were increased among coding DEGs (*r* = 0.44) (**Figure 2B**) but relatively unchanged among noncoding DEGs (*r* = 0.19) (**Figure 2C**). The heatmaps of DEGs among different platforms were shown in **Supplementary Figure S2**.

**Table 2:** Top 10 significant coding and noncoding differentially expressed genes (DEGs) in HTA and their performance on different platforms.

Ensembl gene ID	Gene name	Type	*P*-value	FC	Mega *P*-value	Mega FC	RNA-Seq *P*-value	RNA-Seq FC
Coding								
ENSG00000118113	MMP8	Protein coding	2.16*10^−4^	2.54	1.43*10^−1^	0.63	6.08*10^−1^	1.62
ENSG00000213694	S1PR3	Protein coding	3.36*10^−4^	−1.82	1.97*10^−1^	−0.55	7.91*10^−1^	0.50
ENSG00000124098	FAM210B	Protein coding	4.92*10^−4^	−1.96	5.94*10^−1^	−0.32	9.73*10^−1^	−0.10
ENSG00000012223	LTF	Protein coding	5.38*10^−4^	2.35	6.54*10^−1^	0.51	3.28*10^−1^	−2.53
ENSG00000162711	NLRP3	Protein coding	6.45*10^−4^	−1.94	8.08*10^−2^	−0.72	7.52*10^−1^	−0.79
ENSG00000197822	OCLN	Protein coding	6.54*10^−4^	1.85	8.86*10^−2^	0.57	1.61*10^−1^	4.86
ENSG00000179841	AKAP5	Protein coding	7.21*10^−4^	2.26	2.00*10^−1^	1.01	7.39*10^−2^	6.03
ENSG00000179869	ABCA13	Protein coding	8.78*10^−4^	2.74	1.51*10^−1^	0.63	1.97*10^−1^	3.85
ENSG00000124469	CEACAM8	Protein coding	9.58*10^−4^	2.32	2.94*10^−1^	0.40	8.27*10^−1^	0.58
*^#^ENSG00000127366	TAS2R5	Protein coding	1.66*10^−3^	1.68	2.97*10^−2^	0.61	6.52*10^−2^	7.29
Noncoding								
*ENSG00000223238	RNA5SP294	rRNA	2.30*10^−4^	1.94	2.14*10^−2^	0.89	NA	NA
*ENSG00000259104	PTCSC3	lincRNA	2.39*10^−4^	1.70	9.46*10^−3^	1.45	NA	NA
ENSG00000229278	AL133353.1	Antisense	1.83*10^−3^	1.49	1.37*10^−1^	0.57	9.10*10^−1^	−1.70
ENSG00000253134	AC025437.1	lincRNA	1.89*10^−3^	1.72	1.37*10^−1^	0.63	NA	NA
*ENSG00000212316	RNU6-1228P	snRNA	1.93*10^−3^	1.72	1.01*10^−2^	3.02	9.84*10^−1^	−0.30
ENSG00000231829	AL157834.1	lincRNA	2.03*10^−3^	1.45	3.52*10^−1^	0.41	8.63*10^−1^	−2.57
ENSG00000245105	A2M-AS1	Antisense	2.49*10^−3^	2.65	2.34*10^−1^	0.52	2.58*10^−1^	4.74
ENSG00000231858	AC067945.3	Antisense	2.58*10^−3^	1.64	1.81*10^−1^	0.53	2.51*10^−1^	2.74
ENSG00000251831	RNU6-1114P	snRNA	2.61*10^−3^	−1.42	7.94*10^−1^	−0.13	NA	NA
ENSG00000228139	LINC01402	lincRNA	2.80*10^−3^	1.23	8.02*10^−1^	0.12	4.25*10^−1^	9.41
Cross-platform								
^#^ENSG00000166928	MS4A14	Protein coding	1.83*10^−2^	2.50	1.67*10^−3^	1.52	3.36*10^−2^	4.05
^#^ENSG00000163564	PYHIN1	Protein coding	2.16*10^−2^	1.69	1.10*10^−2^	1.20	4.82*10^−2^	4.14
^#^ENSG00000152818	UTRN	Protein coding	3.09*10^−2^	1.59	2.63*10^−2^	0.78	4.93*10^−2^	4.82
^#^ENSG00000104093	DMXL2	Protein coding	4.55*10^−2^	1.20	1.93*10^−2^	0.68	1.58*10^−2^	6.18

*Significant in HTA and mega-analysis, ^#^validated by quantitative real-time PCR (qRT-PCR).

FC, fold-change; rRNA, ribosomal RNA; snRNA, small nuclear RNA; lincRNA, long intergenic non-coding RNA.

**Figure 3:**
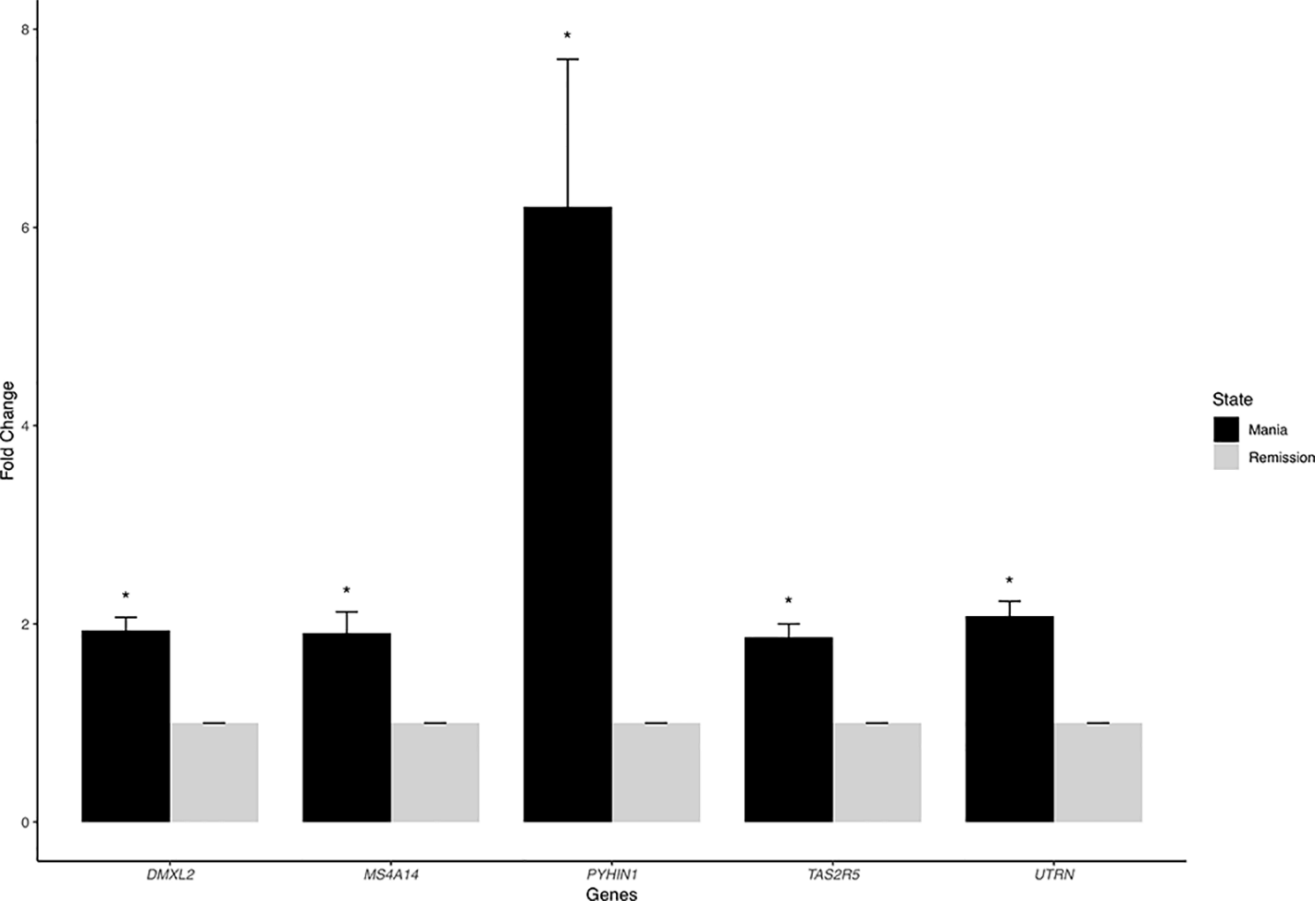
The quantitative real-time PCR (qRT-PCR) results of overlapping DEGs. We chose five overlapping DEGs—MS4A14, PYHIN1, UTRN, DMXL2, and TAS2R5—for validation. All of these genes showed significant differences between manic and remission states with the same direction with original analysis.

We listed the top 10 coding and noncoding DEGs in discovery samples in **Table 2** along with results from other experimental platforms. In general, the expression directions were similar across platforms. Results of the mega-analysis showed that there were more ncRNAs as the DEGs compared with coding DEGs, such as RNA5SP294 (rRNA), PTCSC3 (lincRNA), and RNU6-1228P (snRNA). Among noncoding genes, two miRNAs (*MIR181B1* and *MIR103A1*) showed signals with high levels of gene expression in the manic state (fold change ranged from 1.8 to 3.0). These results of ncRNAs, however, are not able to be examined in our RNA-Seq data (see **Supplementary Figure S3**). We did standard library preparation, which is not designed for obtaining ncRNAs. Thus, most of the noncoding genes were not able to be detected in this platform. For instance, among the DEGs in RNA-Seq analysis, none of the miRNAs were found. Consequently, less noncoding genes were seen in RNA-Seq than microarray platform (**Figure 1C and D**), and in turn, the correlation of fold change in noncoding DEGs was low (see also **Figure 2**). On the other hand, among results of coding genes, we found interesting targets in the taste 2 receptor (TAS2) gene family. The *TAS2R5* and *TAS2R3* genes showed signals in the discovery samples and mega-analysis, suggesting the potential involvement of taste system in the manic state. In general, the DEG targets between microarrays and RNA-Seq did not have much overlapping, and only four genes were significant in microarray and RNA-Seq platforms as well as in mega-analysis: *MS4A14*, *PYHIN1*, *UTRN*, and *DMXL2* (**Table 2**). We used qPCR to validate gene expression patterns of selected gene targets, including the four overlapping genes and *TAS2R5*. All of these genes showed significant differences between manic and remission states in qPCR with the same direction with original analysis (**Figure 3**).

**Figure 4:**
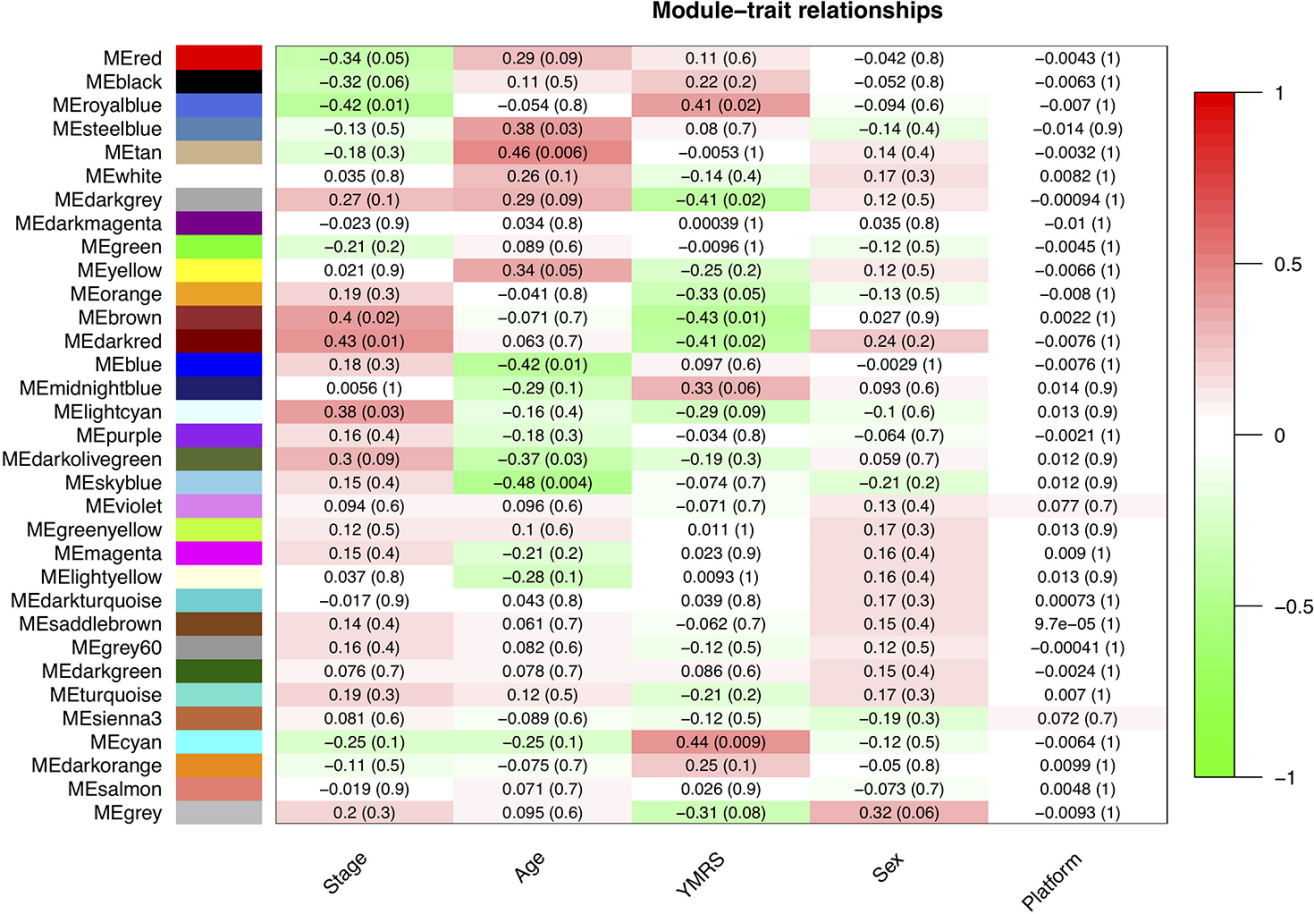
The relationship between 33 modules with clinical traits. The figure was constructed under WGCNA analysis. The correlations between modules and clinical traits were performed with Pearson’s correlation. YMRS: Young Mania Rating Scale; Platform: different array types.

In our network analysis, we found that 34,576 genes were clusterd into 33 modules (median of module size: 324, mean of module size: 1,048; **Figure 4**). There were three modules (Royalblue, Brown, and Darkred) that were significantly correlated with both mania and YMRS score. Two modules (Darkgrey and Cyan) were significantly correlated with YMRS score, and one module (Lightcyan) was significantly correlated with the manic state. We then ran enrichment analysis for these identified modules. In GWAS enrichment analysis, none of the modules were significantly enriched with GWAS signals. For the DEG enrichment analysis, only the Darkred module showed margin significance (*p* = 0.045), and interestingly, all DEGs enriched in this module were ncRNAs (see **Supplementary Table S3**).

Exploration for possible biological functions of each mania-related module was performed, and the results are displayed in **Table 3**. Using the tissue specificity test, we found that Royalblue, Cyan, and Lightcyan modules were enriched in whole blood and Epstein-Barr virus (EBV)-transformed lymphocytes. The genes in Royalblue and Cyan modules were enriched in subtantia nigra (SN) and anterior cingulate cortex (ACC) brain tissue, respectively. In the gene-set enrichment analysis, these two modules were significantly associated with immune-related pathways, including cytokine–cytokine receptor interaction, response to type I interferon and antigen processing, and presentation of exogenous peptide antigen via major histocompatibility complex (MHC) class I.

**Table 3:** Mania-related modules and their enriched possible biological function.

Modules	Traits		Tissue specificity	Gene sets	
	Stage	YMRS	GTEx 53*	Terms	*P*-value
**Royalblue**	⬇	⬆	Lymphocytes	KEGG: Cytokine–cytokine receptor interaction	7.00*10^−3^
			Whole blood	KEGG: Circadian rhythm mammal	7.46*10^−3^
			Substantia nigra	GO: Cytokine-mediated signaling pathway	6.77*10^−4^
				GO: Response to type I interferon	3.97*10^−3^
**Cyan**		⬆	Whole blood	GO: Interferon gamma-mediated signaling pathway	1.95*10^−3^
			Brain BA24	GO: Positive regulation of cytokine production	2.54*10^−3^
				GO: Antigen processing and presentation of exogenous peptide antigen* via* MHC class I	7.01*10^−3^
**Brown**	⬆	⬇	N/A	GO: DNA metabolic process	1.31*10^−3^
				GO: DNA replication	1.31*10^−3^
				GO: Organelle fission	2.08*10^−3^
**Darkred**	⬆	⬇	N/A	N/A	N/A
**Darkgrey**		⬇	Cervix	GO: Hemoglobin metabolic process	8.49*10^−4^
				GO: Negative regulation of actin filament depolymerization	1.31*10^−3^
				GO: ERAD pathway	8.12*10^−3^
**Lightcyan**	⬆		Lymphocytes	N/A	N/A
			Whole blood		
			Heart		
			Cerebellum		

*GTEx 53, The 53 tissue expression from Genotype-Tissue Expression (GTEx); YMRS, Young Mania Rating Scale.

N/A, not applicable; BA24, Brodmann area 24; MHC, major histocompatibility complex; KEGG, Kyoto Encyclopedia of Genes and Genomes; GO, Gene ontology; ERAD, Endoplasmic-reticulum-associated protein degradation.

## Discussion

In the present study, we focused on the manic feature in BD to capture state-specific transcriptome patterns using data from different platforms, and analyzed both coding and noncoding RNAs. Our results were presented by DEG analysis as well as network and enrichment analyses to identify potential transcriptome biomarkers and provide biological explanations. As far as we know, this is the first transcriptome study that reported “state markers” for mania episode with intraindividual comparisons, while previous studies usually compared different groups of patients (22). The interindividual comparisons are subject to confounding bias, such as batch effects, genetic heterogeneity, and different environmental exposures across subjects (20). Therefore, studying dynamic transcriptome changes within individuals is much more preferable in searching biomarkers for the manic state and to provide insights for assisting early diagnosis of BD.

In our DEG analysis, we found that ncRNAs is the predominant category showing mainly up-regulation during acute manic episode (see **Figure 1**), which is possibly related to characteristics of manic symptoms, such as agitation and overenergetic behaviors. Among ncRNAs, the lncRNAs is the largest category in our DEGs. In a previous study, a small number of DEGs were shown for mania, and all of the 22 genes were coding genes. Interestingly, all of them were upregulated in mania (25). Across microarray data (our discovery and German samples), we also found that the fold change is more consistent in ncRNAs than in coding genes; therefore, ncRNAs maybe a relatively reliable targets for manic episode.

In the RNA-Seq results, unfortunately, the original library preparation was not tailored for detecting ncRNAs, and lncRNAs have particularly lower expression than coding genes (48) in non-brain tissues. Thus, most of the ncRNAs reported in our discovery samples cannot be detected by RNA-Seq (**Table 2**). Specific techniques, such as ribosomal RNA depletion for library construction (18) or CaptureSeq (49), are required for more comprehensive ncRNA detection. Similarly, correlations of fold change across microarray and RNA-Seq platforms vary by coding or noncoding category such that the correlations of coding DEGs are higher, ranging from 0.4 to 0.5, and lower for noncoding DEGs (0.2). The lncRNAs showed various regulatory abilities in human genomes (16). Thus, lncRNAs may serve as potential targets for manic episode and could be conveniently assessed by available microarrays. Future studies need to use ribosomal RNA depletion for library construction or CaptureSeq to validate the results of ncRNAs for mania episode biomarkers. Despite our efforts to use multiple datasets to search for more convincing biomarkers for the manic state, none of our DEGs with nominal *p*-value of <0.05 reach statistical significance after multiple testing correction. Our study design with intra-individual comparisons might increase statistical power to a certain extent (50); however, the sample size in the present study might still be too small to detect weak gene effects between acute and remission states. Moreover, heterogeneity in datasets, including ethnics (e.g., Taiwanese and German samples), patients’ inclusion criteria, and clinical characteristics, may also contribute to the increased challenges in finding reliable biomarkers across datasets for the manic state. For the purpose of validation, we selected only the four overlapping DEGs across different platforms and *TAS2R5* (**Table 2**) for experimental validation. All the qPCR results showed significance and high consistency of the fold-change direction in the original analyses, indicating the potential of studying these targets for bipolar illness in future studies. Among the target genes, *MS4A14* belongs to a big gene family called membrane spanning 4-domains. It has been found to be involved in DNA methylation and transcript splicing in Alzheimer’s disease (51). *PYHIN1*, pyrin and HIN domain family member 1, is related to interferon regulation and has been found to be related with depressive behaviors induced by lipopolysaccharide in mice models (52), which showed its potential role as a state marker. *UTRN* encodes for utrophin, which is located at synapse and myotendinous junctions, and was reported to be a candidate gene for schizophrenia and BD (53). An interesting study exploring blood-based spliceosome found that *UTRN* had differential splicing for psychosis in schizophrenia and BD (54), again demonstrating the potential of using blood-based biomarkers for psychiatric disorders. *DMXL2*, Dmx Like 2, is involved in synaptic vesicle exocytosis in major depressive disorder patients (55). *DMXL2* has also been found to be related to co-occurring cardiovascular disease under selective serotonin reuptake inhibitors (SSRI) treatments in patients with major depressive disorder (56). Among the top 10 coding genes reported in the discovery samples, *TAS2R5* is an interesting target that also showed signal in mega-analysis (see **Table 2**). Taste 2 receptors (TAS2Rs) are also called bitter taste receptors, which belong to one of the G protein-coupled receptors. Unlike other TAS2Rs, *TAS2R3* and *TAS3R5* have limited agonist due to their unique structure (57). *TAS2R5* has been found to be overexpressed in asthma children and might have anti-inflammatory function by regulating cytokines (58), indicating its multifunctional characteristic. TAS2Rs are expressed in rat and human brain tissues, which were reported to be related to Parkinson’s disease and neurodevelopmental diseases (59). *TAS2R5* was also found to be downregulated in the dorsolateral prefrontal cortex of schizophrenia postmortem brain tissues compared to healthy control brain tissues (60). Interestingly, another gene, *CES1*, showing signal in our discovery samples (*p* = 7.99*10^−3^) and mega-analysis (*p* = 1.94*10^−2^), was found to be related to taste reduction in attention-deficit/hyperactivity disorder children who underwent methylphenidate treatment (61). In the present study, although the effects of medication were not directly evaluated, we recorded the information for each patient to ensure that medication was not changed during the follow-up period. In clinical observations, the dysregulation of taste often exists during acute episode in BD patients (62). The taste alteration might also be related to cognition performance (63). Therefore, the taste-related receptors are potential candidates for episodic status as the pharmacological targets. Further *in vivo*/*in vitro* experiments are needed to verify the medication effects on these taste-related genes. Recent development of BD patient-derived induced pluripotent stem cells (iPSCs) provides information for medication effects on gene expression (64, 65). For example, recent studies using iPSC of BD patients have produced interesting findings in studying the therapeutic mechanisms of lithium effects (66). This is a potential model to study mechanisms underlying manic state.

For noncoding RNAs among the DEGs, lncRNAs were the predominant category. In our noncoding DEGs, the PTCSC3 (papillary thyroid carcinoma susceptibility candidate 3) gene was reported to be associated with thyroid cancer (67). Researchers nowadays can do a lot of *in silico* prediction for lncRNAs’ functional interpretation (68), and more functions of lncRNA would be validated in the near future. On the other hand, miR181B1 was an interesting target among short ncRNAs, which was upregulated in the manic state. miR181B1 has been found to be downregulated in schizophrenia patients with antipsychotic medication, including olanzapine, quetiapine, ziprasidone, and risperidone (69). Furthermore, another study demonstrates that among treatment-resistant schizophrenia patients, miR181B1 showed higher expression compared to those responsive schizophrenia patients (70). The target genes of miR181B1 might be related to manic symptom onset for BD.

In total, we identified 33 modules by WGCNA with ideal soft threshold (Beta = 4), and 6 modules were significantly related to clinical outcomes (YMRS or manic stage). The mania-related modules were not enriched with GWAS signals even when the signals were from one of the largest BD studies so far (8). The state markers that we explored in the present study might be very different from the susceptible trait loci of BD. From the recent cross-disorder analysis among major psychiatric disorders, schizophrenia, BD, and major depressive disorders (71), the authors reported similar scenario that there exist common genetic loci across different major psychiatric disorders, but transcriptional dysregulation is different.

In the DEG enrichment analysis for modules, we found that most of the modules were not enriched with DEGs, which could be explained by the “omnigeneic model” proposition. This model suggests that the DEGs within the module network are highly connected with other genes, and the biological functions are decided by the whole module but not a few signals. Among the six mania-related modules, we found that the Royalblue and Cyan modules are enriched with gene expression in substantia nigra and BA24 (anterior cingulate cortex) brain regions, which are highly relevant to BD (18, 72). These results supported that peripheral transcriptome analysis can somehow correlated with the dynamic changes in central nervous systems. Moreover, these two modules are especially enriched for cytokine-related immune pathways. These findings are consistent with previous findings for the disturbance of cytokines between different states of BD (73). In addition, among previous gene expression studies based on postmortem brain (17, 18) or iPSCs (19, 74) using case–control study design, most of the enriched pathways were related to calcium channel or G protein-coupled receptors. It is highly possible that dysregulated targets of trait markers or state markers are involved with different sets of genes. These echo the observation of not finding enrichment for GWAS signals in coexpression modules. Information coming from different study designs would provide more comprehensive perspectives in understanding the etiology of bipolar illness. In our results, the cytokine-related pathways are based on genome-wide gene expression network, which might provide more insights for cytokine-related mechanisms underlying manic episode for BD.

There are several limitations in the present study. First, we had limited sample size in the present study, as follow-up of manic BD patients is a difficult challenge. We used different platforms and independent samples for replication and validation, and conducted mega-analysis to increase generalizability. In addition, within-individual paired data are less prone to confounding effects for gene expression study and increase the power for DEG identification. However, the sample size in the present study was small and may not have enough power to detect gene with weak effects. Nevertheless, among our selected gene targets for qPCR validation, all of them showed significant differences with the same expression patterns as the original analyses, which increased our confidence of finding meaningful targets despite moderate power. Second, we used only peripheral blood samples for state-specific transcriptome analysis but not brain tissues for practical reasons. We cannot control the blood drawing time in different subjects and time points, and may slightly influence gene expression results due to potential circadian rhythm in a certain proportion of human gene expressions. Third, the assessment of symptom severity is subject to interviewer bias. Nevertheless, by treating YMRS score as one of the covariates in our linear model, this concern maybe minimized. Lastly, although medication might influence gene expression results, we were not able to control the medications among BD patients in our observational study design, and the medication effects on gene expressions cannot be directly assessed as we are comparing the same individuals at two times points with the same medication treatment during follow-up. Instead, we tried to adjust medication usage of different categories of drugs in regression models, and results remain similar with or without adjustment.

In summary, the present study represents one of the very few studies that focused on manic feature of BD using intraindividual comparisons. Using genome-wide exploration for gene expression patterns with different experimental platforms and sets of independent samples, our results provide the first line of evidence for the involvement of coding and noncoding transcriptomic alterations for manic episode. In particular, with experimental validation, taste-related genes (including *TAS2R5*,* TAS2R3*, and *CES1*) and other common targets across different platforms (*MS4A14*,* PYHIN1*,* UTRN*, and* DMXL2*) might be important targets for episodic onset. Results of network and enrichment analyses suggest the potential role of cytokine-related pathways for mania, and gene expression network is independent of the signals from susceptible genetic loci for BD identified from previous GWA studies. These results bring insights to designing future study for early diagnosis and detection of manic episodes in bipolar illness.

## Ethics Statement 

In the present study, all participants signed informed consent forms after study procedures were fully explained. The sample recruitment and data collection were approved by the Institutional Review Board of all participating institutes and hospitals, including National Taiwan University Hospital Research Ethics Committee, Quality and Patient Safety Committee of Taipei City Hospital and Institutional Review Board of Wangfang Hospital.

## Author Contributions

All investigators contributed to the design or execution of the study, and approved the final version. YC responded to the major data analysis and manuscript writing. PH designed the study, obtained funding, drafted the analytical plan, guided the statistical analysis, interpreted the data, and critically revised the manuscript. MC provided the major parts of resource for data collection and interviewer training. CE, MH, HC, ML, WY and CH responded to interviewer training and data collection. YL and KT conducted the experimental validation. TP and MH provided the resource and guidance for RNA-sequencing techniques.

## Funding

This study was supported by the National Health Research Institutes Project (NHRI-EX106-10627NI), Ministry of Science and Technology Project (MOST 105-2628-B-002-028-MY3), and the National Taiwan University Career Development Project (104R7883) to PI, Dr. PH Kuo, and MOST 107-2314-b-038-085 to PI, Dr. CH Chen, and Taipei City Hospital Research Project (TPCH-108-057) to PI, Dr. MC Huang.

## Conflict of Interest Statement

The authors declare that the research was conducted in the absence of any commercial or financial relationships that could be construed as a potential conflict of interest.
