# Supplementary material for: Transcriptome Changes in Relation to Manic Episode
Source: Front Psychiatry. 2019 May 1;10:280. doi: 10.3389/fpsyt.2019.00280 (PMC6504680; doi:10.3389/fpsyt.2019.00280)
Supplement: Supplementary file 1 [file DataSheet_1.docx]

Supplementary Material

Transcriptome changes in relation to manic episode

Ya-Chin Lee^1^,Yu-Lin Chao^2^, Chiao-Erh Chang^1^, Ming-Hsien Hsieh^3^, Kuan-Ting Liu^1^, Hsi-Chung Chen^3^, Mong-Liang Lu^4,5^, Wen-Yin Chen^6^, Chun-Hsin Chen^4,5^, Mong-Hsun Tsai^7^, Tzu-Pin Lu^1^, Ming-Chyi Huang^5,6,*^, Po-Hsiu Kuo^1,8,*^

^1^Institute of Epidemiology and Preventive Medicine, College of Public Health, National Taiwan University, Taipei, Taiwan

^2^Department of Psychiatry, Buddhist Tzu Chi General Hospital and Tzu Chi University, Hualien, Taiwan

^3^Department of Psychiatry, National Taiwan University Hospital, Taipei, Taiwan

^4^Department of Psychiatry, Wang-Fang Hospital, Taipei Medical University, Taipei, Taiwan

^5^Department of Psychiatry, School of Medicine, College of Medicine, Taipei Medical University, Taipei, Taiwan

^6^Department of Psychiatry, Taipei City Psychiatric Center, Taipei City Hospital, Taipei, Taiwan

^7^Institute of Biotechnology, National Taiwan University, Taipei, Taiwan

^8^Department of Public Health, National Taiwan University, Taipei, Taiwan

*** Correspondence:**Dr. Po-Hsiu Kuo and Dr. Ming-Chyi Huang
E-mail: phkuo@ntu.edu.tw (PH Kuo); mch@tpech.gov.tw (MC Huang)

Keywords: bipolar disorder_1_, manic-episode_2_, microarray_3_, RNA-Sequencing_4_, transcriptome_5_, non-coding RNAs_6_, state-markers_7_

**Supplementary Figure S1 Correlation between p-value of YMRS and Manic Stage under linear model**

In our linear model for DEGs detection, we put YMRS score and Stage in the model. The correlation of p-value between these 2 variable showed that they have highly correlation.

**Supplementary Figure S2 The DEGs heatmap of different platforms**


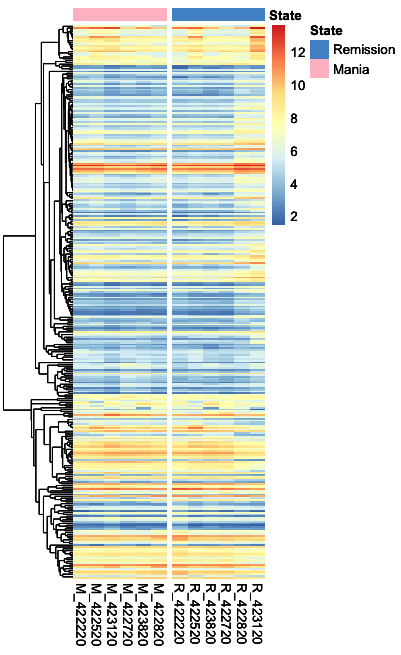

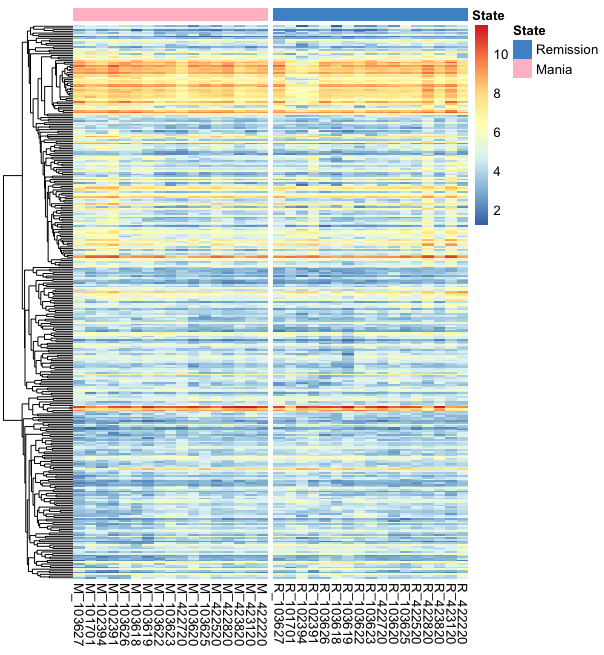

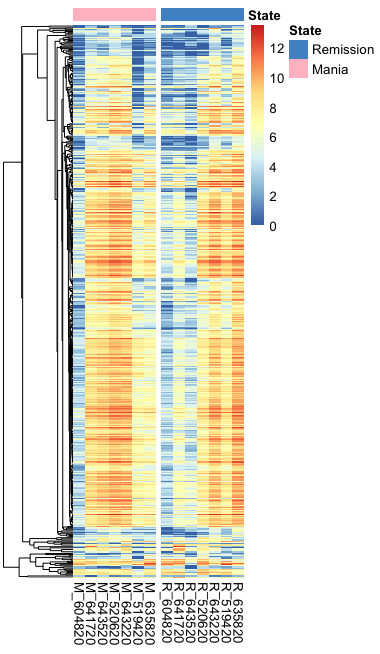


**A**

**B**

**C**

Supplementary Figure S2A showed the heatmap of 306 DEGs among the discovery samples with HTA array.

Supplementary Figure S2B showed the heatmap of 321 DEGs among the mega-analysis

Supplementary Figure S2C showed the heatmap of 491 DEGs among RNA-Seq

**Supplementary Figure S3 The distribution of DEGs in RNA-Seq**

In the DEGs of RNA-Seq, most of the genes belongs to protein-coding genes. The legend of “others” including: pseudogenes, snRNA, snoRNA, process transcript and unclassified.

**Supplementary Table S1 RNA integrity number (RIN) of each samples**

| ID | Manic State | Remission State |
| --- | --- | --- |
| 422220 | 9.4 | 9.6 |
| 422520 | 6.4 | 9.3 |
| 422720 | 9.3 | 9.1 |
| 422820 | 8.9 | 9.7 |
| 423120 | 9.4 | 9.5 |
| 423820 | 9.4 | 8.8 |
| 520620 | 8.8 | 7.9 |
| 519420 | 8.7 | 9.1 |
| 604820 | 9.2 | 9.3 |
| 635820 | 9.4 | 8.5 |
| 641720 | 8.8 | 9.4 |
| 643220 | 8.9 | 8.6 |
| 643520 | 7.8 | 6.3 |

The mean of the total RIN was around 8.8 and there was no significance difference between RIN of manic state and remission state (p>0.05 with pair t-test and wilcoxon signed-rank test).

**Supplementary Table S2 The information of samples for qRT-PCR**

|  | **qRT-PCR Sample** |
| --- | --- |
| **Samples** | 10 |
| **Male (%)** | 5(50) |
| **Age (Mean±SD)** | 44.3±11.2 |
| **YMRS in acute state (Mean±SD)** | 28.1±7.7 |
| **YMRS in remission (Mean±SD)** | 1.6±1.2 |
| **HAMD in acute state (Mean±SD)** | 2.5±1.8 |
| **HAMD in remission (Mean±SD)** | 2.8±1.8 |

**Supplementary Table S3 DEGs and GWAS Enrichment analysis results of manic-related modules**

| **Modules** | **DEGs** | | | **GWAS Signals** | | |
| --- | --- | --- | --- | --- | --- | --- |
|  | **Gene Name** | **Ensemble ID** | **Enrichment P** | **Gene Name** | **Ensemble ID** | **Enrichment P** |
| **Royalblue** | NA | NA | 1 | *SRPK2* | ENSG00000135250 | 5.35*10^-1^ |
| **Brown** | *TAS2R5*  *PPP2R5E*  *CENPS*  *CES1*  AF131215.4  *RNA5SP465* | ENSG00000127366  ENSG00000154001  ENSG00000175279  ENSG00000198848  ENSG00000254936  ENSG00000212497 | 3.89*10^-1^ | *SLC25A17*  *CUL4A*  *NRF1*  *UTP18*  *SP4*  *ADD3*  *VAV3*  *PDE3B* | ENSG00000100372  ENSG00000139842  ENSG00000106459  ENSG00000011260  ENSG00000105866  ENSG00000203876  ENSG00000134215  ENSG00000152270 | 1 |
| **Darkred** | *RNU6-441P*  *MIR4675*  RP1-290I10.3 | ENSG00000207275  ENSG00000265372  ENSG00000228478 | 4.50*10^-2^ | NA | NA | 1 |
| **Darkgrey** | *GATA1* | ENSG00000102145 | 5.06*10^-1^ | NA | NA | 1 |
| **Lightcyan** | *SMG1P5*  *LINC02100* | ENSG00000183604  ENSG00000248693 | 3.71*10^-1^ | *CD47* | ENSG00000196776 | 1 |
| **Cyan** | *MIR147A*  *LTB*  *LINC00377* | ENSG00000207814  ENSG00000227507  ENSG00000229246 | 4.50*10^-1^ | NA | NA | 2.70*10^-1^ |

The DEGs enrichment analysis were conducted with 137 DEGs that significant in primary analysis and meta-analysis also. The GWAS signal were obtained from the latest PGC BD analysis, published on BioRxiv.

**Supplementary Information: The qRT-PCR probes of overlapping DEGs**

| Ensembl Gene ID | Gene Name | Sequence |
| --- | --- | --- |
| ENSG00000166928 | *MS4A14* | F: GATATCCATTCTGGGGAGCA R: TCCGAAGGCAAGAAAAACAG |
| ENSG00000163564 | *PYHIN1* | F: ATCAGCTCCACCCAACACTT R: GCGTAGCCACTGTAGCATGA |
| ENSG00000152818 | *UTRN* | F: AAAGCATTGACGGAAACAGG R: ACTGGAGCTGTAAGGCTGGA |
| ENSG00000104093 | *DMXL2* | F: ATTCCTGGTGCTAAGCATGG R: TTTAAGCCACTGGCACTTGA |
| ENSG00000127366 | *TAS2R5* | F: GAGTCTCTGGTGCCTTCTGG R: AAGGACTTCAGCGCAGTGAT |

**Supplementary Information: The equation of paired sample comparison using limma**

design<-model.matrix(~ID+State+YMRS)

fit<-lmFit(eset, design)

fit<-eBayes(fit)

Here, we followed the tutorial of “limma” (1) to construct the intra-individual comparison model. In the above code: “eset” stood for the normalized expression data of microarray (log 2 expression transformation), “ID” was the sample ID, “State” was the states of the episode (mania or remission) and “YMRS” was the evaluation of symptom severity.

**Reference**

1. Smyth GK. Linear Models and Empirical Bayes Methods for Assessing Differential Expression in Microarray Experiments. *Statistical Applications in Genetics and Molecular Biology* (2004) **3**:1–25. doi:10.2202/1544-6115.1027
